# Dual effects of daily FTY720 on human astrocytes *in vitro*: relevance for neuroinflammation

**DOI:** 10.1186/1742-2094-10-41

**Published:** 2013-03-19

**Authors:** Celina Wu, Soo Y Leong, Craig S Moore, Qiao Ling Cui, Pavel Gris, Louis-Philippe Bernier, Trina A Johnson, Philippe Séguéla, Timothy E Kennedy, Amit Bar-Or, Jack P Antel

**Affiliations:** 1 Montreal Neurological Institute, McGill University, 3801 University St., Montreal QC H3A 2B4, Canada

**Keywords:** Astrocytes, FTY720, Neuroinflammation, Sphingosine-1-phosphate

## Abstract

**Background:**

FTY720 (fingolimod, Gilenya™) is a daily oral therapy for multiple sclerosis that readily accesses the central nervous system (CNS). FTY720 is a structural analog to the sphingolipid sphingosine-1-phosphate (S1P) and is a cognate ligand for the S1P G-protein coupled receptors (S1PR). Studies in experimental autoimmune encephalomyelitis using mice with conditionally deleted S1P_1_R from astrocytes indicate that one beneficial effect of FTY720 in this model is via downregulating external receptors, which inhibits responses induced by the natural ligand. Another proposed effect of FTY720 on neuroinflammation is its ability to maintain persistent signaling in cells via internalized S1P_1_R resulting in functional responses that include suppressing intracellular calcium release. We used human fetal astrocytes to investigate potential dual inhibitory- and function-inducing effects of daily FTY720 on responses relevant to neuroinflammation. For the inhibitory effects, we used signaling and proliferation induced by the natural ligand S1P. For the function-inducing responses, we measured inhibition of intracellular calcium release stimulated by the proinflammatory cytokine, interleukin (IL)-1β.

**Methods:**

Astrocytes derived from human fetal CNS specimens and maintained in dissociated cultures were exposed to 100 nM of the biologically active form of FTY720 over a dosing regimen that ranged from a single exposure (with or without washout after 1 h) to daily exposures up to 5 days. Responses measured include: phosphorylation of extracellular-signal-regulated kinases (pERK1/2) by Western blotting, Ki-67 immunolabeling for cell proliferation, IL-1β-induced calcium release by ratiometric fluorescence, and cytokine/chemokine (IL-6, CXCL10) secretions by ELISA.

**Results:**

We observed that a single addition of FTY720 inhibited subsequent S1PR ligand-induced pERK1/2 signaling for >24 h. Daily FTY720 treatments (3-5 days) maintained this effect together with a loss of proliferative responses to the natural ligand S1P. Repeated FTY720 dosing concurrently maintained a functional cell response as measured by the inhibition of intracellular calcium release when stimulated by the cytokine IL-1β. Recurrent FTY720 treatments did not inhibit serum- or IL-1β-induced pERK1/2. The secretions of IL-6 and CXCL10 in response to IL-1β were unaffected by FTY720 treatment(s).

**Conclusion:**

Our results indicate that daily FTY720 exposures may regulate specific neuroinflammatory responses by desensitizing astrocytes to external S1PR stimuli while sustaining cellular influences that are independent of new surface S1PR activation.

## Background

FTY720 is a clinically approved daily oral therapy used to prevent disease relapses in multiple sclerosis (MS) [1]. FTY720 is a structural analog of the bioactive lipid sphingosine-1-phosphate (S1P) and is a cognate ligand for the G-protein coupled S1P_1, 3, 4, 5_ receptors (S1PR) [1]. The therapeutic effect of FTY720 is currently attributed to the drug’s ability to internalize S1PR (mainly the G_i_-coupled S1P_1_R) on lymphocytes, which results in the cells being unresponsive to the natural ligand S1P, and thus they cannot exit from regional lymph nodes [2].

FTY720 differs from other approved immunomodulatory MS therapies in that it readily accesses the central nervous system (CNS), raising the issue of what functional effects it may have on tissue injury and repair-related processes within the CNS [3]. Intracerebral injections of FTY720 reduced disease severity in the experimental autoimmune encephalomyelitis (EAE) mice independent of systemic lymphopenia [4]. Moreover, systemic administrations of FTY720 to immunodeficient animals enhanced functional recovery following traumatic spinal cord injury [5]; of note is that the effects in both animal models implicate drug interactions with S1PR expressed by astrocytes. It remains unclear, however, if the observed tissue protection/repair processes are the results of inhibiting astrocyte responses to the natural ligand and/or inducing cellular signaling. Previous studies measuring the phosphorylation of extracellular-signal-regulated kinases (pERK1/2) indicate that astrocytes show robust signaling to S1P and FTY720 via S1P_1_R engagement [6,7]. The finding that mice lacking S1P_1_R expression on astrocytes experienced a decreased severity in clinical EAE [8] suggests that a potential benefit of FTY720 on CNS inflammation is by inhibiting extracellular S1P signaling on astrocytes. However, zu Heringdorf et al. demonstrated that activating S1P_1_R in stably transfected (non-neural) cell lines negatively regulates intracellular calcium (Ca^2+^) release and such a release could have a number of neuroinflammation-relevant consequences including mitochondrial stress, production of free radicals, and proteases/phospholipases activation [9,10].

The fate of internalized S1P receptors upon exposure to FTY720 differs from that resulting from interacting with the natural ligand S1P [11]. Receptors internalized consequent to FTY720 binding can persist in intracellular vesicular compartments rather than rapidly recycling to the cell surface as seen with the natural ligand [6]. Using a number of cell lines transfected with S1P_1_R and primary cell types (including rodent astrocytes), Mullershausen et al. showed that signaling by internalized S1P_1_R persists for hours following a single 1-h pulse of FTY720 [6].

Here we applied an experimental regimen of FTY720 on astrocytes derived from the fetal human CNS to model the daily clinical use of the agent. We studied how FTY720 could influence neuroinflammation-relevant responses via its dual role in inhibiting surface S1PR signaling and proliferation while sustaining active responses in the cells as measured by the inhibition of intracellular calcium release when stimulated by the cytokine interleukin (IL)-1β.

## Methods

### Isolation of human fetal astrocytes and cell culture

**Figure 1:**
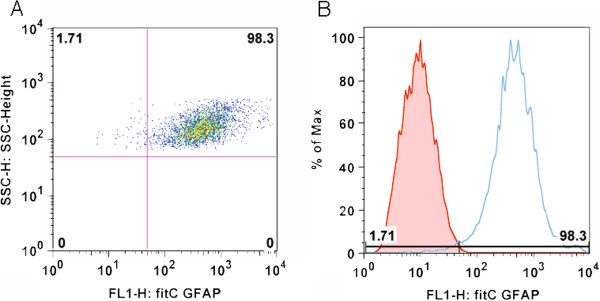
**More than 98% of the cell culture is GFAP positive. **(**A**) Flow cytometry dot plot showing >98% of cells positive for the FITC-labeled GFAP marker. (**B**) Histogram demonstrating the rightward shift for the GFAP + signal compared to isotype control.

CNS tissues were obtained from the human fetal tissue repository (Albert Einstein College of Medicine, Bronx, NY), and experiments were carried out with guidelines approved by McGill University and the Canadian Institutes for Health Research (CIHR). Cells were isolated as previously described by Williams et al. [12]. Briefly, fetal brain tissue (15–18 gestational weeks) was minced and treated with DNase (Roche, Nutley, NL) and trypsin (Invitrogen, Carlsbad, CA) before being passed through a nylon mesh. The flow through was plated at 10^6^ cells/ml in high glucose Dulbecco’s modified essential medium (DMEM; Sigma, Oakville, ON) supplemented with 10% fetal calf serum (FCS) (v/v), penicillin/streptomycin and glutamine (all from Invitrogen, Burlington, ON). Cells were grown in a humidified incubator maintained at 37°C, 5% CO_2_ and passaged every 14 days. To ensure cell purity, all experiments were conducted on the third or fourth passage. More than 98% of cells were GFAP positive as determined by flow cytometry (Figure 1A and B). For serum-free assays, astrocyte media was changed to DMEM-F12 (DMEM; Sigma, Oakville, ON) with 1% bovine serum albumin (Invitrogen, Burlington, ON), penicillin/streptomycin and N1 (GIBCO Life Technologies, Invitrogen, Burlington, ON).

### Pharmaceutical compounds

#### FTY720

FTY720 (2-amino-2-[2-(4-octylphenyl)ethyl]propane-1,3-diol) was provided by Novartis, Basel, Switzerland. In all of the experiments, the phosphorylated form of FTY720 was used. Powdered FTY720 was reconstituted in dimethyl sulfoxide hydrochloric acid (DMSO-HCl) (50mM), aliquoted and stored at −20°C until used. Cells were treated with 100 nM FTY720 in all of the experiments. Initial dose response studies were conducted using FTY720 over a range of 10–1,000 nM (with cell toxicity becoming measurable at the high dose).

#### Sphingosine-1-phosphate

S1P (Sigma, Oakville, ON) was dissolved in 100% methanol, aliquoted and stored at −20°C until use. A final concentration of 100 nM S1P was used in all of the experiments.

#### S1PR-activated pERK1/2 studies

Astrocytes were seeded in six-well plates and cultured to 80% confluency. To assess pERK1/2 responses to FTY720, S1P, IL-1β (10 ng/ml recombinant human IL-1β; PeproTech, Montreal, QC) or 10% FCS (v/v), astrocytes were placed in serum-free DMEM for 6 h and then treated with the stimulus for 15 min. Following stimulation, cells were washed with phosphate-buffered saline (PBS), and whole-cell lysates were collected in 200 μl of RIPA buffer (1% SDS, 1% deoxycholate, 1% Igepal, 150 mM NaCl, 50 mM Tris) with Baculogold protease inhibitor (BD Biosciences, Mississauga, ON) and phosphatase inhibitor (1 mM Na_3_VO_4_; Sigma, Oakville, ON). Samples were stored at −80°C until assayed.

#### Western blotting

Polyvinylidene difluoride membranes were immunoblotted with anti-phospho-ERK1/2 antibody (monoclonal rabbit anti-mouse antibodies 1:1,000; Cell Signaling Technology, Danvers, MA) and detected using horseradish peroxidase-conjugated secondary antibodies (1:1,000; Calbiochem, San Diego, CA) and enhanced chemiluminescence plus reagents (GE Healthcare, Piscataway, NJ). Membranes were then stripped with Reblot (Millipore, Billerica, MA) and re-probed for loading control (anti-β-actin; 1:1,000, Invitrogen or anti-total ERK1/2; 1:1,000 StressGen, Victoria, BC). Protein band intensities were quantified using ImageJ software (National Institutes of Health), and the data reported are expressed as relative fold change to untreated controls.

#### Proliferation assays

A total of 5 × 10^4^ cells per well were plated in 48-well plates. To determine the proportion of astrocytes undergoing mitotic cell division at the time points indicated (see Results), astrocytes were stained with anti-Ki-67 antibody (1:100, rabbit FITC-conjugated; Millipore, Billerica, MA) and with Hoechst dye to label cell nuclei (1:10,000; Invitrogen, Burlington, ON). Images were taken from 16 fields per well and analyzed using MetaXpress cellular imaging analysis software using the cell-scoring module (Molecular Devices, Sunnyvale, CA). Quantitative output measures by MetaXpress processing were used to calculate proliferation rate indices where the proportion of astrocytes positive for Ki-67 was determined as a function of total cell number per field (Hoechst).

#### Effects of FTY720 on pERK1/2 and proliferation studies

In the studies examining the effects of FTY720 on astrocytes signaling and proliferation, FTY720 or S1P was added either at the outset of the culture (with or without washout after 1 h) or daily for 3-5 days. For the pERK1/2-related studies, the above treated cells were re-challenged after 1, 3 or 5 days with FTY720 for 15 min. For the proliferation assays, astrocytes were treated with FTY720 or S1P for 24 h before washout with PBS 3×. S1P was given to astrocytes immediately following washout for an additional 24 h prior to fixing with 4% paraformaldehyde (Sigma, Oakville, ON) and immunostaining for Ki-67.

#### IL-1β Ca^2+^ mobilization studies

To test the whether FTY720 could actively regulate Ca^2+^ mobilization in astrocytes, IL-1β (10 ng/ml recombinant human IL-1β, dissolved in Ca^2+^-free PBS; PeproTech, Montreal, QC) was used as a stimulus for Ca^2+^ release from internal stores. Astrocytes were plated at a high density of 2 × 10^5^ cells/ml in 96-well plates and cultured in DMEM-F12 medium for up to 5 days under the different FTY720 exposure regimens outlined previously in the signaling-related (pERK1/2) studies. Following FTY720 treatment(s), astrocytes were washed with Ca^2+^-free PBS and subsequently loaded with a calcium-sensitive fluorescent dye fura-2 AM (5μM, Molecular Probes) suspended in Ca^2+^-free Ringer’s solution (130 mM NaCl, 3 mM KCl, 1 mM MgCl_2_, 10 mM HEPES, pH 7.4) for 45 min in a humidified chamber at 37°C, 5% CO_2_. Cells were washed 2× with Ca^2+^-free PBS to remove extracellular fura-2 AM and incubated in Ca^2+^-free Ringer’s solution for an additional 30 min at room temperature to allow complete hydrolysis of acetoxymethyl esters before imaging. The Wallac Victor^3^ (Perkin-Elmer, Wellesley, MA) fluorescent microplate reader was used to measure ratiometric intracellular Ca^2+^ concentrations. IL-1β was loaded in the integrated injector and delivered at a volume of 25 μl/well. To establish baseline values, five fluorescent ratio measurements were taken before the IL-1β injection, and 30 subsequent recordings were made immediately following IL-1β delivery into each well. Data reported represent means from three independent experiments with each observation averaged over at least eight wells per condition. Fluorescence emission ratios are expressed as intracellular Ca^2+^ values using the formula described by Grynkiewicz et al. [13]. Magnitudes of response (to the IL-1β agonist) were calculated by measuring the difference between peaks of Ca^2+^ curves with baseline values.

#### Cytokine/chemokine production

To assess whether FTY720 exposure(s) on astrocytes affect their productions of cytokines/chemokines (in response to IL-1β stimulation) and whether FTY720 itself induces IL-6 and CXCL10 (IP-10), astrocytes were treated with FTY720 ± IL-1β. For the + IL-1β conditions, astrocytes were stimulated with IL-1β for 24 h before collecting supernatants. The levels of IL-6 and CXCL10 present in supernatants were measured in duplicates using ELISA plates following the manufacturer’s instructions (BD Biosciences, Mississauga, ON).

#### Statistical analyses

Statistical analyses were performed with Prism 5 (GraphPad Software). One-way analysis of variance (ANOVA) with Bonferroni post hoc tests was used to compare mean values. Significance was accepted at the *p* < 0.05 level (*p < 0.05; **p < 0.01; ***p < 0.001). The number of individual studies performed for each set of experiments is indicated in the Results section and in figure legends.

## Results

### S1P receptor-dependent effects of FTY720 and S1P

For these studies, we measured pERK1/2 activation and proliferation responses following either a single dosage of FTY720 or repeated daily administrations.

### Single exposure studies

#### pERK1/2 activation

**Figure 2:**
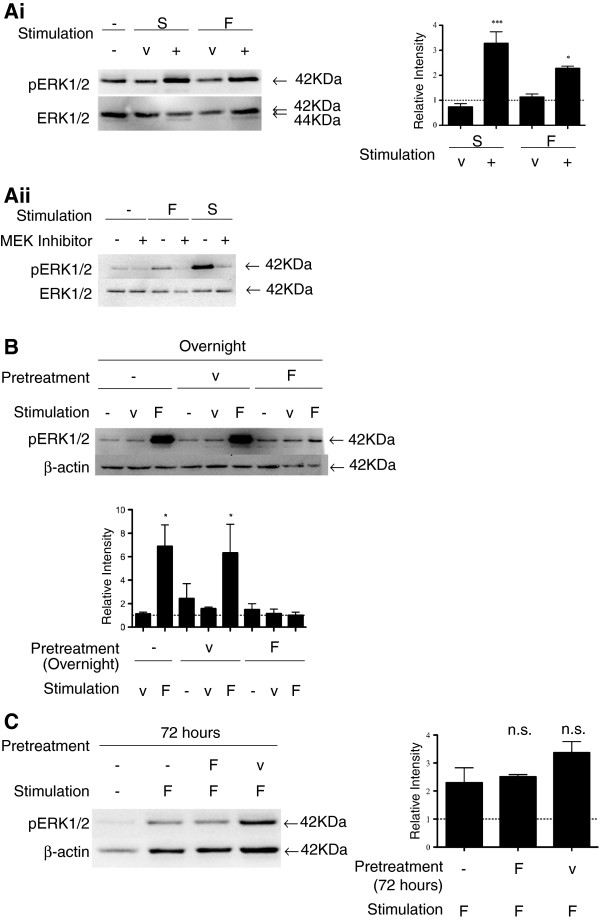
**(A) Human fetal astrocytes respond to FTY720 or S1P exposure by signaling through the ERK1/2 pathway. (i)** Astrocytes were exposed to FTY720 or S1P for 15 min. Western blot: untreated *(−)*; S1P vehicle (*v*); S1P *(S)* (100 nM); FTY720 vehicle (*v*); FTY720 *(F)* (100 nM). S1P and FTY720 induced significant pERK1/2 at 15 min. Total ERK1/2 was used as the loading control. Quantified band intensity relative to untreated control (*n* = 3). (**ii**) Activation signals by FTY720 and S1P are dependent on MEK1/2. MEK inhibitor was applied to astrocytes with FTY720 or S1P for 15 min. Western blot lanes: *1,* untreated *(−)*; *2,* MEK inhibitor; *3,* FTY720 (*F*); *4,* FTY720 plus MEK inhibitor; *5,* S1P (*S*); *6,* S1P plus MEK inhibitor. The MEK inhibitor itself did not induce notable pERK1/2. When the MEK inhibitor was applied with FTY720 or S1P, pERK1/2 signals induced by either ligand were comparable to the untreated control. Total ERK1/2 was used as the loading control. (**B**) *FTY720 treatment overnight inhibits pERK1/2 activation by subsequent FTY720 exposure.* FTY720 *(F)* (15 min) induced significant pERK1/2 in untreated *(−)* astrocytes and those pre-treated overnight with the vehicle (*v*). Pre-treating astrocytes with FTY720 overnight resulted in a blunted pERK1/2 signal upon re-challenge with FTY720 (15 min). β-Actin was used as the loading control. Quantified band intensity relative to untreated control (*n* = 3). (**C**) *Recovery of pERK1/2 response at 72 h following initial FTY720 exposure.* FTY720 (*F*) (15 min) induced significant pERK1/2 in untreated astrocytes *(−)* and those pre-treated with a single dose of FTY720 or vehicle (*v*) for 72 h. Recovery of pERK1/2 activation by FTY720 was achieved by 72 h following initial FTY720 treatment. β-Actin was used as the loading control. Quantified band intensity relative to untreated control (*n* = 3).

As shown in Figure 2Ai (and in Additional file 1), pERK1/2 signaling was evident in astrocytes exposed to FTY720 or S1P at 15 min, as previously reported in Durafourt et al. [14] and Mullershausen et al. [6]. ERK1/2 phosphorylation induced by either FTY720 or S1P was blocked by adding 10 μM of the mitogen-activated protein kinase/ERK kinase (MEK) inhibitor U0126 (Promega) (Figure 2Aii). When astrocytes were incubated overnight (18 h) with an initial dose of FTY720, the intensity of pERK1/2 signaling evoked by a new 15-min FTY720 challenge was reduced compared to cells maintained in serum-free culture medium (Figure 2B). Similar reductions were noted when S1P was used as stimulus (15 min) (Additional file 2). Cells cultured with S1P overnight showed a pERK1/2 response comparable to control cells when challenged with FTY720 (Additional file 3). Figure 2C shows the inhibited pERK1/2 response by FTY720 pre-exposure fully recovered by 72 h following initial treatment.

#### S1P-induced proliferation

**Figure 3:**
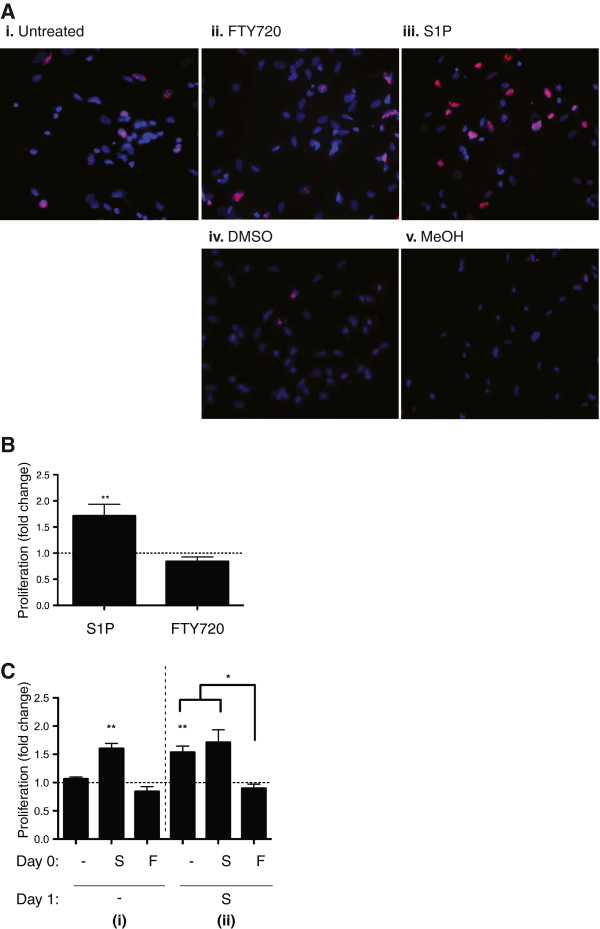
**(A) Human fetal astrocyte proliferation in response to S1P receptor ligands.** Immunocytochemistry stains for astrocytes undergoing cell division (Ki-67, *red*) and cell nuclei (Hoechst nuclear stain, *blue*). (**i**) Untreated cells (basal proliferation). (**ii**) FTY720 (100 nM) overnight. (**iii**) S1P (100 nM) overnight. (**iv**) DMSO control. (**v**) Methanol control (MeOH). (**B**) *Quantification of human fetal astrocyte proliferation in response to S1P receptor ligands*. Proliferation indices were generated by determining the percentage of cells positive for Ki-67 relative to total cell population (Hoechst nuclear stain). Fold changes in proliferation were calculated relative to rate of proliferation under basal conditions. S1P induced a 1.8-fold increase in astrocyte proliferation, whereas FTY720 did not mediate proliferation beyond the basal rate (normalized to 1) (*n* = 3). (**C**) *FTY720 pre-incubation blocks proliferative response of S1P on human fetal astrocytes.* At outset (Day 0), astrocytes were either treated with S1P (*S*) or FTY720 (*F*). Following overnight culture, cells were either (*i*) left untreated *(−)* or (*ii*) incubated with S1P for another 24 h (Day 1). Proportion of astrocytes undergoing proliferation (Ki-67+) was then determined. Hoechst nuclear stain was used to determine total cell number. Proliferation fold change was calculated relative to rate of proliferation under basal conditions (normalized to 1). (**i**) S1P induced a 1.6-fold increase in astrocyte proliferation whereas FTY720 was comparable to basal proliferation rate. (**ii**) Significant proliferation (1.5-fold increase) was observed in untreated astrocytes (Day 0) when given S1P for 24 h (Day 1). S1P-pre-treated astrocytes on Day 0 continued to proliferate (1.8-fold increase), whereas FTY720 exposure on Day 0 did not result in astrocyte proliferation when exposed to S1P stimulation added on Day 1 (*n* = 3).

As illustrated in Figure 3A (and quantified in Figure 3B), S1P overnight elicited a 1.8-fold increase in astrocyte proliferation as measured by the percentage of cells positive for Ki-67 (nuclear protein, marker for proliferation [15]). Astrocytes incubated overnight with FTY720 did not produce a similar proliferation effect. Figure 3C shows the proliferation rates of astrocytes to S1P when pre-exposed with S1P or FTY720 overnight (Day 0). Initial Day 0 treatment with S1P increased astrocyte proliferation (1.6-fold increase), whereas FTY720 was comparable to basal proliferation rates (i). Subsequent (Day 1) S1P stimulation for 24 h increased astrocyte proliferation in cells maintained in culture medium alone (1.5-fold increase) or pre-treated with S1P (ii). Astrocytes exposed to FTY720 overnight on Day 0 did not demonstrate a proliferative response to the S1P given on Day 1.

The above results suggest that a single treatment with FTY720 desensitizes cell surface S1P receptors for >24 h.

### Extended treatment studies

#### pERK1/2 signaling

**Figure 4:**
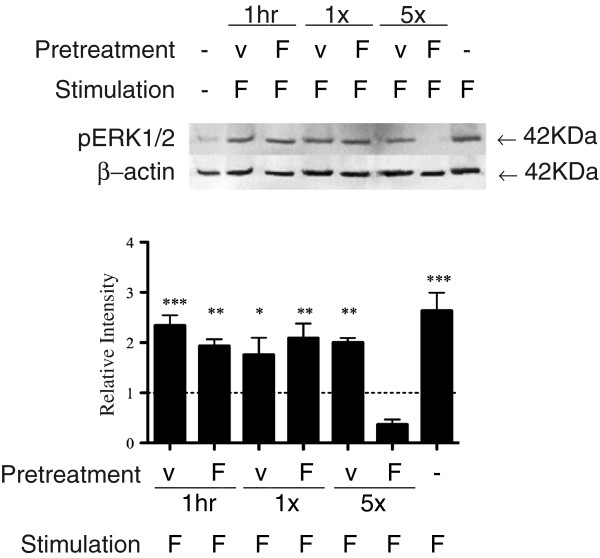
**Repeated (daily) FTY720 administration sustains the inhibition of pERK1/2 response.** pERK1/2 levels in response to FTY720 (*F*) (100 nM) for 15 min in astrocytes that were initially exposed to a single dose of FTY720 (*F*) or vehicle (*v*) with washout after 1 h (1 h), or without washout (1×) or repeated daily for 5 days (5×). pERK1/2 activation by FTY720 was observed in the 1-h and single without-washout conditions, but not in the repeated daily FTY720 condition. β-Actin was used as the loading control. Quantified band intensity relative to untreated control (*n* = 3).

As shown in Figure 4, astrocytes treated with FTY720 daily for 5 days (or 3 days, Additional file 4A) showed a reduced pERK1/2 response to the FTY720 challenge (15 min) compared to astrocytes maintained in culture medium alone (untreated control). pERK1/2 signals in astrocytes treated with FTY720 only at the initiation of cell culture (either left in or washed after 1 h) were comparable to the untreated controls. Addition of serum to astrocyte cultures treated once or repeatedly with FTY720 provoked a robust increase in pERK1/2, indicating the preserved integrity of this signaling pathway in the cells (Additional file 4B).

These above results suggest that repeated daily treatment with FTY720 sustains desensitization of cell surface S1P receptors.

#### Functional effects of FTY720

**Figure 5:**
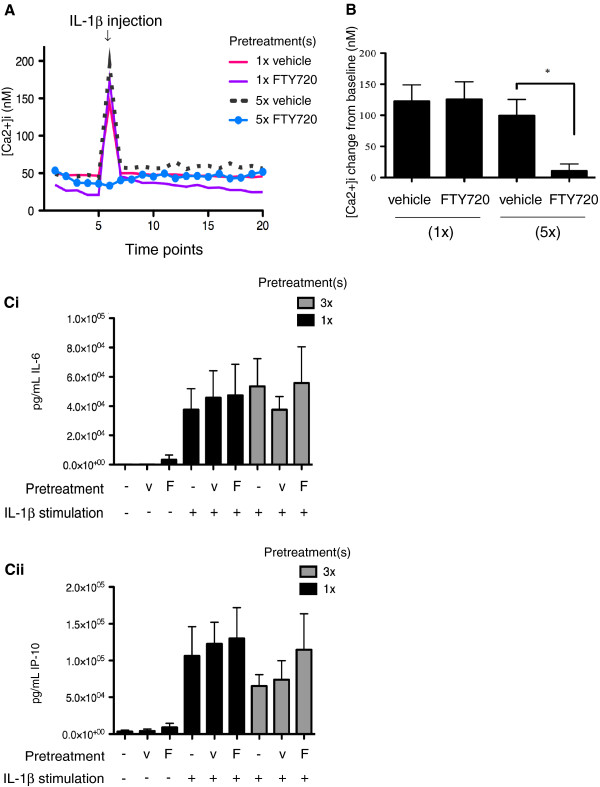
**(A) Repeated FTY720 administrations inhibit IL-1β-induced Ca**^**2+ **^**mobilization in human fetal astrocytes.** Representative traces showing daily FTY720 for 5 days inhibited IL-1β (10 ng/ml)-induced Ca^2+^ mobilization; this effect was not seen with FTY720 (1× FTY720) or vehicle (1× vehicle) added only at outset of the culture, nor with the vehicle control added daily for 5 days (5× vehicle). (**B**) Bar graph showing the mean (±SEM) changes in Ca^2+^ mobilization under conditions described in (**A**). *Signifies comparison between FTY720 (5×) and vehicle (5×) (*n* = 3). (**C**) *Pre-treatment of human fetal astrocytes with FTY720 does not alter IL-6 or CXCL10 (IP-10) secretions induced by IL-1β.* Astrocytes pre-treated with either FTY720 (*F*) or with the vehicle (*v*) control at outset of culture (1×) or daily for three cycles (3×). After the 72-h culture, IL-1β (10 ng/ml) was added and cells were cultured for an additional 24 h before collecting supernatants (*n* = 3). (**i**) Astrocytes produced significant levels of IL-6 in response to IL-1β activation. Levels of IL-6 produced were not affected by FTY720 pre-treatment(s), and FTY720 itself did not induce significant IL-6 secretion. (**ii**) Astrocytes produced significant levels of CXCL10 (IP-10) response to IL-1β activation. Pre-treating astrocytes with FTY720 did not alter CXCL10 secretion induced by IL-1β, and FTY720 itself did not induce CXCL10 secretion (*n* = 3).

To assess the capacity of FTY720 to mediate a functional effect in astrocytes, we examined inhibition of Ca^2+^ mobilization in these cells. Figure 5A presents the magnitude of Ca^2+^ mobilization in astrocytes stimulated with IL-1β. As shown in Figure 5A and B, repeated daily treatments with FTY720 (e.g., the condition that desensitizes S1P receptors) were able to abrogate the Ca^2+^ efflux induced by IL-1β stimulation. There was no inhibition of IL-1β-induced Ca^2+^ mobilization after 5 days in astrocyte cultures exposed to a single dose of FTY720 given at the outset. However, an apparent partial inhibition of Ca^2+^ release was observed at the initial overnight time point following exposure to FTY720 (Additional file 5). Neither FTY720 nor S1P themselves induced significant Ca^2+^ release in astrocytes when compared to their vehicles (changes from baseline were: FTY720 468 ± 193 nM versus vehicle 942 ± 434 nM; S1P 463 ± 171 nM versus vehicle 443 ± 94 nM, *n* = 5 separate experiments). As shown in Figure 5Ci and Cii, FTY720 added either once or daily for 3 days did not significantly block IL-1β-induced productions of IL-6 or CXCL10 (IP-10), and FTY720 itself did not stimulate IL-6 or CXCL10 release. Furthermore, FTY720 did not affect the IL-1β-induced pERK1/2 responses (Additional file 6).

These results indicate that repeated daily applications of FTY720 mediate specific functional responses in astrocytes even when cell surface receptors are desensitized.

## Discussion

In the current study, we demonstrate that repeated daily FTY720 administrations can evoke dual effects on astrocytes, both of which may be relevant to the modulation of neuroinflammatory responses by this compound. We initially show that FTY720 desensitizes responses that are dependent on surface S1PR signaling including FTY720 and S1P ligand-induced ERK1/2 phosphorylation and S1P-induced proliferation. We did not observe astrocyte proliferation with FTY720 even though astrocyte proliferation is attributed to S1P_1_R activation [7,16,17] and existing data suggest that FTY720 predominantly activates S1P_1_R [18]. Astrocyte proliferation is a histological feature of active neuroinflammatory conditions [reviewed in Pekny and Nilsson (2005)] and would be predicted to be associated with the production of multiple molecules that regulate or mediate inflammatory responses [19].

Our observations follow up on the studies of Mullershausen et al. who utilized labeled receptors transfected in cell lines to track receptor localization (cell membrane vs. internalized) after exposure to FTY720 or S1P [6]. Their study showed that at 5 h after exposure to FTY720, the S1P receptors are internalized and plasma membrane-dependent signaling responses to FTY720 or S1P are reduced (referred to as *receptor desensitization*) [6]. They showed that under such conditions there was persistent signaling via the internalized receptors [6]. We now show that daily FTY720 maintains this dual effect of desensitizing membrane-dependent signaling while permitting internal receptor-dependent responses.

Differential processing of internalized S1P receptors by FTY720 versus the natural ligand S1P is well described in cell line transfection studies using labeled receptors [11,20]. Oo et al. found that FTY720 results in vesicular storage of receptors prior to ubiquitination and degradation, whereas S1P induces rapid re-cycling to the cell surface [11]. We now demonstrate that repeated daily application of FTY720 maintains this surface receptor desensitization, which results in the loss of both pERK1/2 activation to external FTY720/S1P and proliferation to S1P. Such astrocytes, however, do retain their capacity to respond to non-S1PR-mediated stimuli and signal through the ERK1/2 pathway as shown by the robust pERK1/2 response to serum and IL-1β.

In the absence of surface S1PR-dependent signaling and proliferation, we demonstrate that FTY720 can continue to exert a functional response in human astrocytes. As mentioned, FTY720 mainly binds S1P_1_R [7,16,17], and zu Heringdorf et al. have previously shown that ongoing S1P_1_R stimulation inhibited the ATP-evoked calcium release by activating PKCα and PKCβI, negative-regulators of PLC [21]. We measured the extent of intracellular calcium inhibition in cells treated with FTY720 and used the proinflammatory cytokine IL-1β to stimulate the release of intracellular calcium stores in such cells. This calcium-inhibitory effect persisted when FTY720 was applied daily over the course of 5 days, whereas astrocytes given a single application of FTY720 at the outset of treatment responded to IL-1β by releasing significant levels of calcium. IL-1-receptor activation on astrocytes leads to signaling through the NF-kB pathway and triggers the release of calcium from intracellular stores [21-23]. Activation of calcium-signaling pathways in response to IL-1β stimulation could have a number of functional consequences, including mitochondrial stress, production of free radicals and proteases/phospholipases activation [10]. The inhibition of calcium release by daily FTY720 treatments did not impair astrocytes’ production of the cytokine IL-6 or the chemokine CXCL10 (IP-10) in response to IL-1β. While both FTY720 and S1P have been reported to increase intracellular calcium levels [6], we did not observe direct calcium mobilization from cultured astrocytes in response to either of the ligands.

## Conclusion

In this study, we investigated the functional effects of repeated daily doses of FTY720 on human fetal astrocytes *in vitro*. We showed the potential of daily FTY720 in desensitizing astrocytes from surface receptor-dependent signaling by measuring pERK1/2 activation and proliferation induced by the natural ligand S1P. We also showed that daily FTY720 sustained an inhibition effect on calcium release upon IL-1β stimulation. Collectively, our data indicate that FTY720 can mediate dual neuroinflammation-relevant effects on astrocytes by inhibiting external S1P receptor activation while sustaining internal S1PR-signaling influences.

## Abbreviations

Ca2+: Calcium; CNS: Central nervous system; CXCL10: C-X-C motif chemokine 10; FTY720: 2-amino-2-[2-(4-octyl-phenyl)ethyl]-1, 3-propanediol hydrochloride; HFA: Human fetal astrocytes; IL-1β: Interleukin-1 beta; IL-6: Interleukin-6; IP-10: Interferon gamma-induced protein 10; MS: Multiple sclerosis; pERK1/2: Phospho-extracellular signal-regulated kinases1/2; PKC: Protein kinase C; PLC: Phospholipase C; S1P: Sphingosine-1-phosphate; S1PR: Sphingosine-1-phosphate receptor.

## Competing interests

The authors declare that they have no competing interests.

## Authors’ contributions

CW: Designed and conducted the research experiments and prepared the manuscript. SYL: Was involved in the analysis and interpretation of data and revised the manuscript for intellectual content. CSM: Was involved in the analysis and interpretation of data and revised the manuscript for intellectual content. QLC: Prepared the MAPK/ERK inhibitor samples and acquired the immunocytochemistry data related to FTY720 induction of pERK1/2 activation. PG: Was involved in the analysis and interpretation of data and revised the manuscript for intellectual content. LPB: Was involved in the acquisition of the calcium data and revised the manuscript for intellectual content. TAJ: Provided insight to the conception of the project and revised the manuscript for intellectual content. PS: Provided intellectual feedback and revised the manuscript for intellectual content. TEK: Provided intellectual feedback and revised the manuscript for intellectual content. ABO: Provided intellectual feedback, supervised the study and revised the manuscript for intellectual content. JPA: Supervised all aspects of the project, drafted the manuscript and revised it for intellectual content. All authors read and approved the final manuscript.

## Supplementary Material

Additional file 1 ** (A-D) Untreated; (E-H) vehicle; (I-L) FTY720 (100 nM).** Scale bar = 10 μm. Merged images show the colocalization of pERK (*red*) signal with GFAP + (*green*) cells. Most abundant pERK signal is observed in the FTY720-treated condition. Nuclear DAPI stain (*blue*). Click here for file

Additional file 2 **FTY720 treatment overnight inhibits pERK1/2 signaling by subsequent S1P stimulation.** S1P (*S*) (100 nM) and FTY720 *(F)* (100 nM) stimulation for 15 min induced significant pERK1/2 in untreated *(−)* astrocytes. Pre-treating astrocytes with FTY720 overnight resulted in a blunted pERK1/2 signal upon re-exposure to either FTY720 or S1P for 15 min. Total ERK1/2 was used as the loading control. Click here for file

Additional file 3 **S1P treatment overnight does not inhibit pERK1/2 activation by subsequent FTY720 exposure.** FTY720 (*F*) (100 nM) stimulation for 15 min induced significant pERK1/2 in untreated *(−)* astrocytes and in astrocytes pre-treated overnight with S1P (*S*) (100 nM). β-Actin was used as the loading control. Click here for file

Additional file 4 **(A) Repeated (daily) FTY720 administration for 3 days sustains the inhibition of pERK1/2 response.** pERK1/2 levels in response to FTY720 (*F*) (100 nM) for 15 min in astrocytes that were initially exposed to a single dose of FTY720 with washout after 1 h (1h), or without washout (1×), or repeated daily for 3 days (3×). pERK1/2 activation by FTY720 was observed in the 1-h and the single without washout conditions but not in the repeated daily FTY720 condition. β-Actin was used as the loading control. (**B**) FTY720 treatment does not inhibit pERK1/2 induction by serum. pERK1/2 levels in response to 10% fetal calf serum for 15 min in astrocytes that were initially exposed to a single dose of FTY720 (1×) or repeated daily for 3 days (3×). pERK1/2 activation by 10% serum was observed in all of the pre-treatment conditions. β-Actin was used as the loading control. Click here for file

Additional file 5 **FTY720 overnight treatment inhibits IL-1β-induced Ca**^**2+ **^**mobilization in human fetal astrocytes.** Overnight treatment with FTY720 (100 nM) inhibited IL-1β (10 ng/ml)-induced Ca^2+^ mobilization compared to vehicle control. Click here for file

Additional file 6 **S1P or FTY720 treatment overnight does not affect IL-1β activation of pERK1/2.** IL-1β (10 ng/ml) stimulation for 15 min induced significant pERK1/2 in untreated *(−)* astrocytes and in those pre-treated overnight with S1P (*S*) (100 nM) or FTY720 (*F*) (100 nM). Total ERK1/2 was used as the loading control. Click here for file
