# Supplementary figures and images for: Dual effects of daily FTY720 on human astrocytes in vitro: relevance for neuroinflammation
Source: J Neuroinflammation. 2013 Mar 19;10:41. doi: 10.1186/1742-2094-10-41 (PMC3621211; doi:10.1186/1742-2094-10-41)

Additional file 1

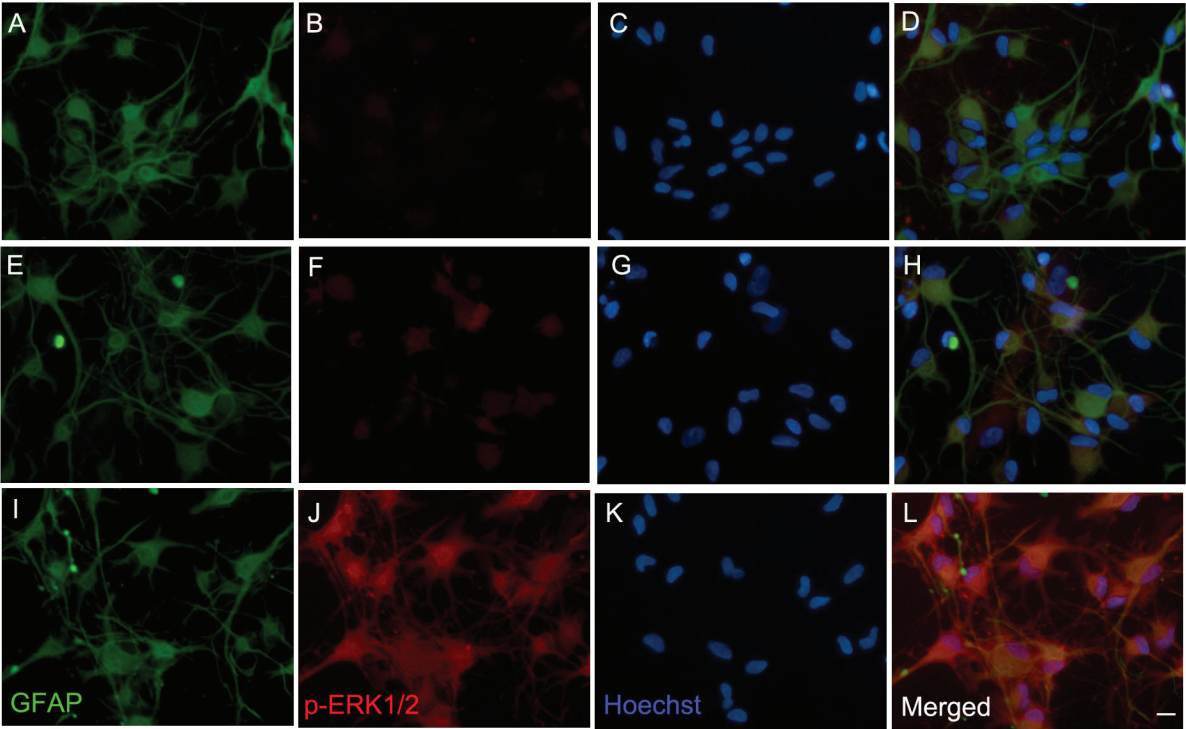

Supplement: Additional file 1 — (A-D) Untreated; (E-H) vehicle; (I-L) FTY720 (100 nM). Scale bar = 10 μm. Merged images show the colocalization of pERK (red) signal with GFAP + (green) cells. Most abundant pERK signal is observed in the FTY720-treated condition. Nuclear DAPI stain (blue). [file 1742-2094-10-41-S1.pdf]

Additional file 2

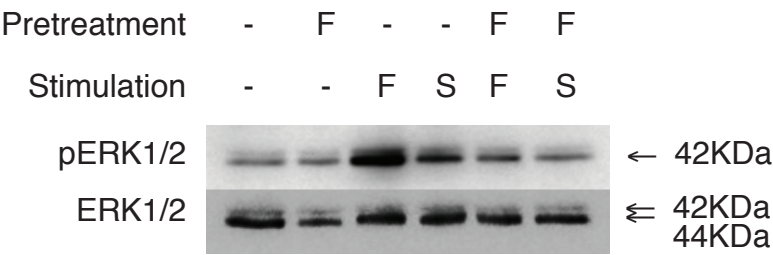

Supplement: Additional file 2 — FTY720 treatment overnight inhibits pERK1/2 signaling by subsequent S1P stimulation. S1P (S) (100 nM) and FTY720 (F) (100 nM) stimulation for 15 min induced significant pERK1/2 in untreated (−) astrocytes. Pre-treating astrocytes with FTY720 overnight resulted in a blunted pERK1/2 signal upon re-exposure to either FTY720 or S1P for 15 min. Total ERK1/2 was used as the loading control. [file 1742-2094-10-41-S2.pdf]

**Additional file 3**

|              |   |   |   |   |
|--------------|---|---|---|---|
| Pretreatment | - | S | - | S |
| Stimulation  | - | - | F | F |

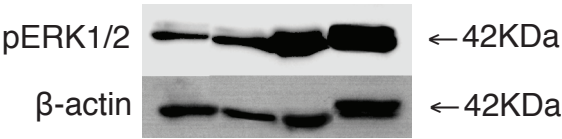

Supplement: Additional file 3 — S1P treatment overnight does not inhibit pERK1/2 activation by subsequent FTY720 exposure. FTY720 (F) (100 nM) stimulation for 15 min induced significant pERK1/2 in untreated (−) astrocytes and in astrocytes pre-treated overnight with S1P (S) (100 nM). β-Actin was used as the loading control. [file 1742-2094-10-41-S3.pdf]

**Additional file 4a**

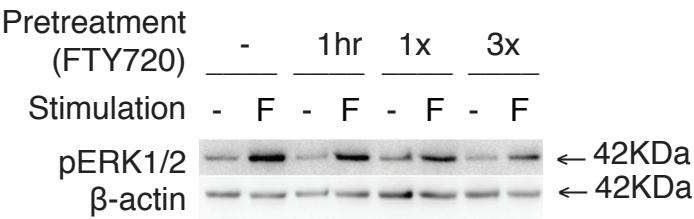

**Additional file 4b**

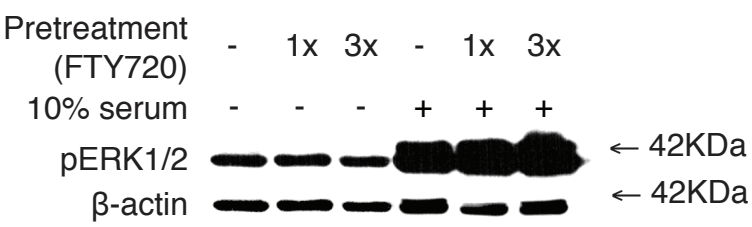

Supplement: Additional file 4 — (A) Repeated (daily) FTY720 administration for 3 days sustains the inhibition of pERK1/2 response. pERK1/2 levels in response to FTY720 (F) (100 nM) for 15 min in astrocytes that were initially exposed to a single dose of FTY720 with washout after 1 h (1h), or without washout (1×), or repeated daily for 3 days (3×). pERK1/2 activation by FTY720 was observed in the 1-h and the single without washout conditions but not in the repeated daily FTY720 condition. β-Actin was used as the loading control. (B) FTY720 treatment does not inhibit pERK1/2 induction by serum. pERK1/2 levels in response to 10% fetal calf serum for 15 min in astrocytes that were initially exposed to a single dose of FTY720 (1×) or repeated daily for 3 days (3×). pERK1/2 activation by 10% serum was observed in all of the pre-treatment conditions. β-Actin was used as the loading control. [file 1742-2094-10-41-S4.pdf]

Additional file 5

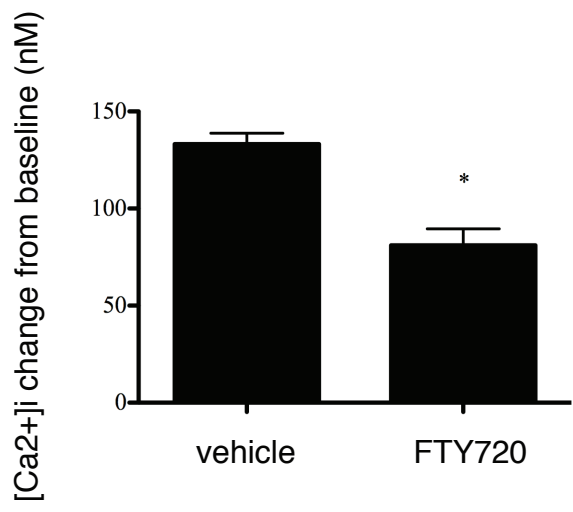

Supplement: Additional file 5 — FTY720 overnight treatment inhibits IL-1β-induced Ca2+ mobilization in human fetal astrocytes. Overnight treatment with FTY720 (100 nM) inhibited IL-1β (10 ng/ml)-induced Ca2+ mobilization compared to vehicle control. [file 1742-2094-10-41-S5.pdf]

Additional file 6

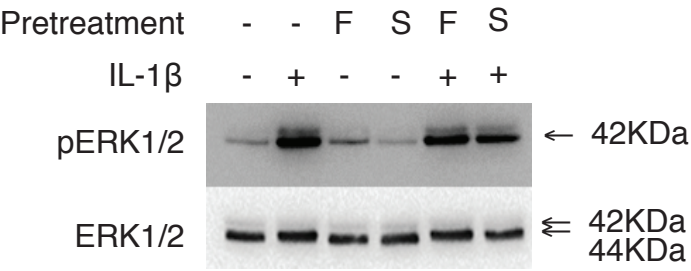

Supplement: Additional file 6 — S1P or FTY720 treatment overnight does not affect IL-1β activation of pERK1/2. IL-1β (10 ng/ml) stimulation for 15 min induced significant pERK1/2 in untreated (−) astrocytes and in those pre-treated overnight with S1P (S) (100 nM) or FTY720 (F) (100 nM). Total ERK1/2 was used as the loading control. [file 1742-2094-10-41-S6.pdf]
